# Supplementary material for: Evaluation of a mobile-based remote aftercare application in cochlear implant users
Source: PLoS One. 2026 Feb 3;21(2):e0326650. doi: 10.1371/journal.pone.0326650 (PMC12867254; doi:10.1371/journal.pone.0326650)
Supplement: S1 File — (PDF) [file pone.0326650.s001.pdf]

Age	Gender - Selected Choice	Hearing loss	Hearing Solution on the left side	How many hours per day do you use a hearing solution on the left side?	When was the first fitting of your hearing implant on the left side? Month - Month - First Fitting (left ear)	When was the first fitting of your hearing implant on the left side? Month - Year - First Fitting (left ear)	Hearing Solution on the right side	How many hours per day do you use a hearing solution on the right side?
	Male	Left side,Right side	Cochlear Implant (CI)	>12	8	2014	Cochlear Implant (CI)	>12
41-50 years	Female	Left side,Right side	Cochlear Implant (CI)	>12	1	2013	Cochlear Implant (CI)	>12
	Female	Right side					Cochlear Implant (CI)	10-dic
	Female	Right side					Cochlear Implant (CI)	10-dic
	Female	Left side	Cochlear Implant (CI)	>12	5	2018		
<15 years	Male	Left side,Right side	Cochlear Implant (CI)	>12	2	2013	Cochlear Implant (CI)	10-dic
15-20 years	Female	Left side,Right side	Cochlear Implant (CI)	>12	11	2014	Cochlear Implant (CI)	>12
21-30 years	Female	Left side,Right side	Cochlear Implant (CI)	>12	9	2004	Hearing Aid	>12
<15 years	Female	Left side,Right side	Cochlear Implant (CI)	>12	7	2014	Cochlear Implant (CI)	>12
<15 years	Male	Left side,Right side	Cochlear Implant (CI)	10-dic	10	2022	Cochlear Implant (CI)	10-dic
<15 years	Male	Left side,Right side	Cochlear Implant (CI)	>12	1	2014	Cochlear Implant (CI)	>12
<15 years	Female	Right side					Cochlear Implant (CI)	07-sep
21-30 years	Female	Left side,Right side	Cochlear Implant (CI)	>12	10	2004	Hearing Aid	>12
41-50 years	Female	Left side,Right side	Hearing Aid	<1			Cochlear Implant (CI)	>12
41-50 years	Male	Left side,Right side	Cochlear Implant (CI)	04-jun	5	2021	Hearing Aid	04-jun
51-60 years	Male	Left side	Cochlear Implant (CI)	>12	3	2022		
41-50 years	Female	Left side,Right side	Hearing Aid	01-mar	5	2010	Cochlear Implant (CI)	07-sep
>70 years	Female	Left side,Right side					Cochlear Implant (CI)	10-dic
>70 years	Female	Left side,Right side	Hearing Aid	>12	12	2000	Cochlear Implant (CI)	>12
>70 years	Male	Left side,Right side	Hearing Aid	>12	3		Cochlear Implant (CI)	10-dic
>70 years	Male	Left side,Right side	Cochlear Implant (CI)	>12	4	2011		

61-70 years	Male	Left side,Right side					Cochlear Implant (CI)	>12
<15 years	Male	Left side,Right side	Cochlear Implant (CI)	>12	8	2014	Cochlear Implant (CI)	>12
61-70 years	Male	Left side,Right side					Cochlear Implant (CI)	>12
41-50 years	Male	Left side,Right side					Cochlear Implant (CI)	>12
21-30 years	Female	Left side,Right side	Cochlear Implant (CI)	>12	8	2004		
21-30 years	Male	Left side,Right side	Cochlear Implant (CI)	>12	4	2015		
21-30 years	Male	Left side,Right side	Cochlear Implant (CI)				Cochlear Implant (CI)	>12
61-70 years	Male	Right side					Cochlear Implant (CI)	>12

When was the first fitting of your hearing implant on the right side? Month - Month - First Fitting (left ear)	When was the first fitting of your hearing implant on the right side? Month - Year - First Fitting (left ear)	How do you agree with the following statements?<br><br>Please rate the following statements on the scale from 1 (strongly disagree) to 7 (strongly agree). - RemoteCheck allows me to save money	How do you agree with the following statements?<br><br>Please rate the following statements on the scale from 1 (strongly disagree) to 7 (strongly agree). - I would recommend RemoteCheck to others	Ease of use and satisfaction<br><br>1. RemoteCheck was easy to use.	Ease of use and satisfaction<br><br>2. It was easy for me to learn to use RemoteCheck.	Ease of use and satisfaction<br><br>3. I like the interface of RemoteCheck.	Ease of use and satisfaction<br><br>4. The information in RemoteCheck was well organized, so I could easily find the information I needed.	Ease of use and satisfaction<br><br>5. I feel comfortable using RemoteCheck in social settings.	Ease of use and satisfaction<br><br>6. The amount of time involved in using RemoteCheck has been fitting for me.
7	2016	6	6	7	7	6	6	5	5
1	2015	7	7	6	7	7	7	6	6
5	2017	4		5	7	5	5		4
5	2017	4		6	6	5			5
		2	3	3	4	3	3	3	3
5	2015	7	7	7	5	7	5	6	7
11	2014	1	1	7	7	7	7	7	7
9	2003	7	7	6	6	5	6		4
2	2016	5	7	7	6	7	7	7	7
1	2015			7	7	7	7	6	7
11	2015	7							
6	2017								
6	2003	5	5	7	7	6	5		
6	2022	7	7	6	7	6	7	6	6
1	2016	2	6	4	4	4	6	5	5
		7	7	7	7	5	6	6	6
4	2022	7	7	7	7	7	5		7
1	2022	6	6	6	4	5	6	5	6
5	2022	7	7	7	7	7	7		7
10	2021	6	4	4	4	4	5	4	5
		4	6	7	5	6	6		7

11	2019	6	6	6	6	6	6	5	6
7	2016	5	6	6	6	6	6	6	6
		7	7	7	7	1	7	1	7
10	2018	7	6	6	7	7	6	5	6
		6	6	6	7	7	6	6	5
		7	7	6	6	6	7	7	6
7	2003	7	7	7	7	7	7	7	7
3	2015	7	7	7	7	7	7	7	7

Ease of use and satisfaction<br><br>7. I would use RemoteCheck again.	Ease of use and satisfaction<br><br>8. Overall, I am satisfied with RemoteCheck.	System information arrangement<br><br>9. Whenever I made a mistake using RemoteCheck, I could recover easily and quickly.	System information arrangement<br><br>10. RemoteCheck provided an acceptable way to receive health care services.	System information arrangement<br><br>11. RemoteCheck adequately acknowledged and provided information to let me know the progress of my action.	System information arrangement<br><br>12. The navigation was consistent when moving between screens.	information arrangement<br><br>13. The interface of RemoteCheck allowed me to use all the functions (such as entering information, responding to reminders, viewing information) offered by RemoteCheck.	System information arrangement<br><br>14. RemoteCheck has all the functions and capabilities I expect it to have.	Usefulness<br><br>15. RemoteCheck would be useful for my health and well-being.	Usefulness<br><br>16. RemoteCheck improved my access to health care services.
6	6	5	5	5	5	6	6	6	6
6	6				6			6	
4		1			4			4	4
4	4	2						3	3
3	4	4	4	4	4	4	4	4	4
7	7	7	7	6	7	6	7	7	4
7	7	6	6	7	7	6	6	7	7
6	6				6			5	
7	7	5	7	7	7	7	7	6	7
7	7								
5	5		4	4	6			4	
7	7	3	7	7	7	7	7	6	6
5	5	6	5	5	6	6	6	6	6
6	6	4	6	6	6	5	6	6	6
7	7	7			7	7	7	7	7
5	6	6	4		6			4	4
7	7				7			7	
4	4	5	5	5	6	4	4	4	4
6	6	5	6	6	6	6	6	7	6

6	6	6	6	6	6	6	6	6	6
5	5	5	6	6	5	5	5	5	5
7	1	7	7	7	7	7	7	7	7
7	6	6	6	6	7	7	6	5	5
6	6	6	6	6	6	6	6	6	6
7	7							7	6
7	7	7	7	7	7	7	7	7	7
7	7	7	7	7	7	7	7	7	7

Usefulness<br><br>17. RemoteCheck helped me manage my health effectively.	Usefulness<br><br>18. RemoteCheck made it convenient for me to communicate with my health care provider.	Usefulness<br><br>19. Using RemoteCheck, I had many more opportunities to interact with my health care provider.	Usefulness<br><br>20. I felt confident that any information I sent to my provider using RemoteCheck would be received.	Usefulness<br><br>21. I felt comfortable communicating with my health care provider using RemoteCheck.	Do you visit a CI audiologist?	If you do not see an CI audiologist what is the reason? - Selected Choice	What is the approximate distance in kilometres to your CI audiologist?	How much time to you need to factor in for one visit to your CI audiologist (incl. travel time, being with your professional, etc.)?	How much money do you need to factor in for one visit to your audiologist (e.g. fuel, hotel, parking, etc.)?
6	6	6	4	6	Once a year		> 100 km	5 - 6 hrs	51-100 €
		7	7	7	Once a year		> 100 km	7 - 8 hrs	51-100 €
2	4		4	4	Once a year		41 - 70 km	3 - 4 hrs	<50 €
2	3	3			Once a year		41 - 70 km	3 - 4 hrs	<50 €
3	3	5	3	5	Once a year		> 100 km	3 - 4 hrs	<50 €
5	6	6	7	7	Once a year		41 - 70 km	3 - 4 hrs	<50 €
7	7	7	7	7	Once a year		21 - 40 km	3 - 4 hrs	<50 €
					Once a year		> 100 km	More than 8hrs but less than a day	51-100 €
7	7	7	7	7	Once a year		5 - 10 km	3 - 4 hrs	<50 €
					Once a year		> 100 km	5 - 6 hrs	51-100 €
					Every 6 months		41 - 70 km	3 - 4 hrs	<50 €
			5	5	Once a year		> 100 km	More than 8hrs but less than a day	> 150 €
6	7	7	7	7	Every 6 months		21 - 40 km	Less than 2hr	<50 €
6	6	6	6	6	Every 6 months		> 100 km	3 - 4 hrs	<50 €
6	6	5	7	6	Every 6 months		> 100 km	Less than 2hr	<50 €
7	7	7	7	7	Every 6 months		41 - 70 km	3 - 4 hrs	<50 €
4	4		4	4	Every 6 months		5 - 10 km	Less than 2hr	<50 €
		7		7	Every 6 months		5 - 10 km	Less than 2hr	<50 €
4	4	4	4	5	Every 6 months		5 - 10 km	Less than 2hr	<50 €
6	6	6	6	7	Once a year		11 - 20 km	Less than 2hr	<50 €

5	6	6	6	6	Once a year		> 100 km	3 - 4 hrs	> 150 €
5	5	5	6	6	Once a year		71 - 100 km	5 - 6 hrs	51-100 €
7	7	7	7	7	Once a year		<5 km	Less than 2hr	<50 €
5	6	7	7	7	Once a year		> 100 km	3 - 4 hrs	51-100 €
6	6	6	6	6	No	I don't feel the necessity			
6	7	6	7	7	Once a year		21 - 40 km	Less than 2hr	<50 €
7	7	7	7	7	Once a year		<5 km	Less than 2hr	<50 €
7	7	7	7	7	Less than once a year		> 100 km	5 - 6 hrs	101-150 €

Do you need support by someone to visit your CI audiologist?	If you go to see your CI audiologist, do you need to take a day off from school/university/work?	Do you visit an ENT specialist/CI surgeon?	If you do not see an ENT specialist/CI surgeon what is the reason? - Selected Choice	What is the approximate distance in kilometers to your ENT specialist/CI surgeon?	How much time to you need to factor in for one visit to your ENT specialist/CI surgeon (incl. travel time, being with your professional, etc.)?	How much money do you need to factor in for one visit to your ENT specialist/CI surgeon (e.g. fuel, hotel, parking, etc.)?	Do you need support by someone to visit your ENT specialist/CI surgeon?	If you go to see your ENT specialist/CI surgeon, do you need to take a day off from school/university/work?
No	Unpaid leave	Once a year		> 100 km	5 - 6 hrs	51-100 €	No	Unpaid leave
Yes	Unpaid leave	Once a year		> 100 km	7 - 8 hrs	51-100 €	Yes	Unpaid leave
Yes	Paid leave	Once a year						
Yes	Paid leave	Once a year		41 - 70 km	3 - 4 hrs	< 50 €	Yes	Paid leave
Yes	Paid leave	Once a year		> 100 km	3 - 4 hrs	< 50 €	Yes	Paid leave
Yes	Paid leave	No	I don't feel the necessity					
Yes	Unpaid leave	Once a year		21 - 40 km	3 - 4 hrs	< 50 €	Yes	Unpaid leave
No	Unpaid leave	Once a year		> 100 km	More than 8hrs but less than a day	51-100 €	No	Unpaid leave
Yes		Once a year		5 - 10 km	3 - 4 hrs	< 50 €	Yes	
Yes	Unpaid leave	Once a year		> 100 km	5 - 6 hrs	51-100 €	Yes	Unpaid leave
Yes	Paid leave	Every 6 months		41 - 70 km	3 - 4 hrs	< 50 €	Yes	Paid leave
No		Once a year		> 100 km	More than 8hrs but less than a day	> 150 €	No	
No	Paid leave	No	I don't feel the necessity					
No	No	Once a year		> 100 km	3 - 4 hrs	< 50 €	No	No
No	Paid leave	Every 6 months		> 100 km	Less than 2hr	< 50 €	No	No
Yes	No	Every 6 months		41 - 70 km	3 - 4 hrs	< 50 €	Yes	No
Yes	No	No	Other					
Yes	Unpaid leave	Once a year		<5 km	Less than 2hr	< 50 €	Yes	Unpaid leave
No	No	Once a year		<5 km	Less than 2hr	< 50 €	No	No
Yes	No	No	Other					

No	No	No	Other					
No	Paid leave	Once a year		11 - 20 km	Less than 2hr	< 50 €	No	No
Yes	No	Once a year		<5 km	Less than 2hr	< 50 €	Yes	No
No	Unpaid leave	No	I don't feel the necessity					
		No	I don't feel the necessity					
No	Unpaid leave	Once a year		21 - 40 km	Less than 2hr	< 50 €	No	Unpaid leave
Yes	Paid leave	No						
Yes	Unpaid leave	Less than once a year		> 100 km	5 - 6 hrs	101-150 €	Yes	Paid leave

Are you doing rehabilitation with a professional?	If you do not see a rehabilitation specialist what is the reason? - Selected Choice	What is the approximate distance in kilometers to your rehabilitation specialist?	How much time to you need to factor in for one visit to your rehabilitation specialist (incl. travel time, being with your professional, etc.)?
No	Other		
Multiple times a month		11 - 20 km	3 - 4 hrs
No	I don't feel the necessity		
Multiple times a month		5 - 10 km	Less than 2hr
Multiple times a month		11 - 20 km	Less than 2hr
Once a year		21 - 40 km	3 - 4 hrs
No	I don't feel the necessity		
Multiple times a month		5 - 10 km	3 - 4 hrs
Multiple times a month		11 - 20 km	3 - 4 hrs
No	Other		
No	I don't feel the necessity		
No	I don't feel the necessity		
No	Other		
Every 6 months		> 100 km	Less than 2hr
Every 6 months		41 - 70 km	3 - 4 hrs
No	Other		
No			
No	Other		
No	Other		

No	Other
No	I don't feel the necessity
No	Other
No	I don't feel the necessity
No	I don't feel the necessity
No	Other
No	
No	Other

How much money do you need to factor in for one visit to your rehabilitation specialist (e.g. fuel, hotel, parking, etc.)?	Do you need support by someone to visit your rehabilitation specialist?	If you go to see your rehabilitation specialist, do you need to take a day off from school/university/work?	Satisfaction	Interface	Usefulness	TotalScoreSatisfaction	AvgScoreSatisfaction	TotalScoreInterface	AvgScoreInterface
			48	32	40	48	6	32	5,33
< 50 €	Yes	No	51	6	27	51	6,38	6	6
			30	5	22				
			30	2	14	30	5	2	2
< 50 €	Yes	No	26	24	27	26	3,25	24	4
< 50 €	Yes	No	51	40	42	51	6,38	40	6,67
< 50 €	Yes	Unpaid leave	56	38	49	56	7	38	6,33
			39	6	5	39	5,57	6	6
< 50 €	Yes		55	40	48	55	6,88	40	6,67
			55	0	0				
< 50 €	No	No	0	0	0	0		0	
			0	0	0	0		0	
			35	14	14	35	5,83	14	4,67
			52	38	46	52	6,5	38	6,33
			38	34	42	38	4,75	34	5,67
< 50 €	No	Paid leave	49	33	42	49	6,13	33	5,5
< 50 €	Yes	No	47	28	49	47	6,71	28	7
			43	16	24	43	5,38	16	5,33
			49	7	21	49	7	7	7
			34	29	29	34	4,25	29	4,83
			43	35	44	43	6,14	35	5,83

47	36	41	47	5,88	36	6
46	32	37	46	5,75	32	5,33
38	42	49	38	4,75	42	7
50	38	42	50	6,25	38	6,33
49	36	42	49	6,13	36	6
52	0	46	52	6,5	0	
56	42	49	56	7	42	7
56	42	49	56	7	42	7

TotalScoreUsefulness	AvgScoreUsefulness	TotalScore	AvgTotalScore
40	5,71	120	40
27	6,75	84	28
14	2,8	46	15,33
27	3,86	77	25,67
42	6	133	44,33
49	7	143	47,67
5	5	50	16,67
48	6,86	143	47,67
0		0	0
0		0	0
14	4,67	63	21
46	6,57	136	45,33
42	6	114	38
42	6	124	41,33
49	7	124	41,33
24	4	83	27,67
21	7	77	25,67
29	4,14	92	30,67
44	6,29	122	40,67

41	5,86	124	41,33
37	5,29	115	38,33
49	7	129	43
42	6	130	43,33
42	6	127	42,33
46	6,57	98	32,67
49	7	147	49
49	7	147	49
