# Supplementary material for: Evaluation of a mobile-based remote aftercare application in cochlear implant users
Source: PLoS One. 2026 Feb 3;21(2):e0326650. doi: 10.1371/journal.pone.0326650 (PMC12867254; doi:10.1371/journal.pone.0326650)
Supplement: Attachment — Submitted filename: Review Report_Plosone.docx [file pone.0326650.s002.docx]

**Reviewer’s comments**

General

The present study objective is to compare clinical follow-up sessions using the MEDEL remote check application with conventional face-to-face follow-up sessions. The specific domains are usability and user friendliness of the platform from CI users/caregivers/clinicians, time and costs involved, and comparing the diagnosis made through two modalities (remote control and face-to-face sessions). I appreciate the authors for making an effort to assess the use of a remote check web-based application in the MEDEL cochlear device. There are section-specific comments to be addressed and looked into, which might help the author to improve the visibility of the manuscript.

**Introduction**

1. The literature review is linked with the need for the study. However, an extensive literature review about the parameters focused on by the author in this manuscript is missing. Therefore, authors should include studies to provide better understanding.
2. The author mentioned that “*Follow-up sessions typically entail a visit by the user to a specialist at an audiology center. This can pose several challenges and inconveniences*.” This needs supporting literature in specific to the challenges faced by the patient/caregivers apart from those mentioned in this paragraph. I feel the author should mention and differentiate the mandatory visit to the hospital versus remote consultation, since this depends upon the duration of the implant use. Once a patient's device is switched on, they need more frequent visits, and it's essential to have face-to-face consultation, whereas after years of device use, their issues and consultation can be taken care of through remote control.
3. In line 78-79, it is mentioned as “Ideally, routine follow-up sessions could take place remotely with the consultation and speech perception testing carried while the CI user was at home”. Can the author be specific about the speech perception testing possible to perform in the remote control mode?
4. In lines 82-85, it is mentioned as “MED-EL Remote Check is a web-based application which is designed to facilitate remote assessment of aided hearing performance and to collect patient reported outcome measures (PROMs) implemented as questionnaires.” , The author should emphasize after how many months of the implant age or device uses, MEDEL remote check web based application can help gather the required information. Further, whether the questionnaire is targeted to the patient/caregivers/children/adults should be mentioned with a little more detail for better understanding of the readers.

**Method**

1. In the method, it's mentioned as a multi-center, open-label, open-arm, prospective study. However, the data collection is done only from two centers.
2. In line 216-227, The details about the number of participants and other demographic details (unilateral/bimodal/bilateral, children, adult, pre-lingual/post-lingual) should be mentioned in the method chapter rather than in the results section. It is not clear why authors consider different duration of experience across different professionals (ENT/Audiologist/SLT), which probably influences the findings.
3. In line 163-164, it is mentioned that “A forward and back-translation approach was used to produce a Spanish translation of the instrument”, is this validated on the Spanish individuals. If yes, authors can mention the same.

**Results**

1. The information about the participants must be shifted in the method section.
2. What was the method adopted by the author to collect data on 29 CI users? Was this data sample appropriate to make inferences?
3. There are 29 CI users, but the data shown in the MAUQ response is of only 27 CI users. What about the remaining two participants?
4. In Table 2, only the mean is mentioned instead median and SD also needs to be added.
5. In user survey, again data is only about 28 CI users though the total participants are 29. Furthermore, there is a lot of variability observed among the participants.
6. Under the professional survey, there are only 3 participants who participated, which limits the generalization of the findings.
7. The statistics used are only descriptive. There are no inferential statistics across the conditions, measures used to strengthen the finding.

**Discussion**

1. The summary of the findings mentioned in the discussion section is actually a repetition of the information and is not essentially needed. It might not provide any additional understanding to the reader.
2. The author mentioned that “The results of the present study are broadly in accord with this, in that users demonstrated motivation to use the application and found it to be usable.”. How the present study findings are in line of the study done by Philips et al (2018). Did the author evaluate the motivation aspects during the remote check app application?
3. The discussion section written at present is, in general, rather specific to the findings of the present study or, the domain explored in the present study.
4. The author added the study limitations and which makes readers cautious while implementing the findings of the study.

Overall, I observe certain limitations that need to be addressed. Therefore, I recommend a major revision before considering the manuscript in its present form.
